# Quality assessment and variety classification of seed‐used pumpkin by‐products: Potential values to deep processing

**DOI:** 10.1002/fsn3.1276

**Published:** 2019-11-19

**Authors:** Qinqin Chen, Ying Lyu, Jinfeng Bi, Xinye Wu, Xin Jin, Yening Qiao, Haonan Hou, Chunmao Lyu

**Affiliations:** ^1^ Institute of Food Science and Technology Chinese Academy of Agricultural Sciences (CAAS) /Key Laboratory of Agro‐Products Processing Ministry of Agriculture and Rural Affairs Beijing China; ^2^ Department of Food Science Shenyang Agricultural University Shenyang China

**Keywords:** by‐products, E‐nose, principal component analysis, seed‐used pumpkin, system cluster analysis

## Abstract

Seed‐used pumpkin (SUP) is known as a traditional popular crop, which is mainly processed for seeds. However, the by‐products (49 times the amount of seeds) were disposed directly into the field as waste. In this study, potential values of seed‐used pumpkins’ by‐products (SUPBs, peel and pulp) as food resource were investigated. Physico‐chemical, nutritional, and aroma profile of ten varieties of SUPBs were characterized, and variety differences were also distinguished. Peel “a*” value, water, fructose, crude fat, sucrose, and Ca contents were the 6 characteristic indicators of SUPBs which screened through correlation analysis, principal component analysis (*PCA*), and *PCA‐X* model. Comprehensive evaluation of physico‐chemical, nutritional, and aroma profile, four varieties by‐products (Jf8#, Nf8#, Rbf#, and Rf9#) were always characterized into Cluster Ⅰ. Other varieties were classified into Cluster Ⅱ based on aroma profile. However, two varieties by‐products (Db1# and Xn1#) presented significant differences from others (Db2#, Db3#, Db4#, and Myxc2#) in physico‐chemical and nutritional indices, they were classified as Cluster III and IV, respectively. Db1# had the highest nutritional value of soluble solid (11.78 ºBx), pectin (1,166.15 mg/ 100 g), total carotenoid (19.57 mg/ 100 g), and total sugar (13.69 g/ 100 g). Among all the SUPBs, Db1# had a relatively higher nutritional value, which was suitable as food resource for deep processing.

## Graphical abstract

Comprehensive quality of SUPB was first investigated systematically. Six characteristic indicators were screened to represent the whole quality of SUPB. Ten varieties of SUPB were mainly divided into two clusters, and Db1# had a higher nutritional value which could be used as potential food resource.

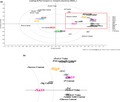

## 1 INTRODUCTION

Seed‐used pumpkin (SUP) is known as a traditional popular crop, with an incredibly rich and long history (Kates, Soltis, & Soltis, 2017; Smith, 1997) and is mainly processed for seeds. Nowadays, SUPs are mainly grown in Asia, and *Cucurbita moschata*, *C. pepo* as well as *C. maxima* are the main species which are cultivated in the spring, available during the fall. In China, the planting area of SUP is about 350,000 hectares, and the annual output of seeds is over 150,000 tons which accounts for 70% of the annual world exports of pumpkin seeds Wei, Chen, Yin, and Jing (2013).

Pumpkin seeds and seed oil are often made into products for human consumption (Liu et al., 2013). Since the natural high contents of some unsaturated fatty acids were determined in the products (Kita, Kucharska, Sokół‐Łętowska, Biesiada, and Nawirska‐Olszańska 2013; Rabrenović, Dimić, Novaković, & Tešević, 2014), their good oxidation resistance and nutritional value had been proved by lots of studies (Xanthopoulou, Nomikos, Fragopoulou, & Antonopoulou, 2009 Eraslan, Kanbur, Aslan, & Karabacak, 2013; Wang et al., 2017). As a consequence, the demand of SUPs had been increasing at China and abroad in the past few decades, and more and more attention was paid on high‐quality production of SUPs and pumpkin seeds (Jing, 2006; Ren, Hao, & Ma, 2011; Xu & Zhao, 2012). However, some unavoidable problems encountered in the industry. As calculated in our study, peel and pulp of SUP were the primary waste by‐products, and one ton of SUP could generate approximately 49 times of by‐products than the amount of seeds. While 85% of the by‐products were discarded directly into the field as waste, except for 15% of which for animal feed or as organic fertilizers. It was not only environment‐unfriendly but also reduced the utilization of the SUP. Moreover, by‐products of SUP may include a rich but yet underutilized source of valuable compounds which could be further used as food additives or dietary supplements (Genevois, Flores, & Escalada, 2016). However, few studies have been carried out to evaluate the potential value of seed‐used pumpkins’ by‐products (SUPBs).

Ten common varieties of SUPs which are widely grown in China are chosen in this study. Physio‐chemical, nutritional, and aroma profiles of SUPBs were evaluated, and characteristic evaluation indicators were screened through multivariate statistical analysis including correlation analysis (*CA*), principal component analysis (*PCA*), and *PCA‐X* model analysis. The variety differences of SUPBs were distinguished by *PCA*, linear discriminant analysis (*LDA*), and system cluster analysis (*SCA*).

## 2 MATERIALS AND METHODS

### 2.1 Raw materials

Ten varieties of SUPs were chosen as raw materials including five varieties of *C. maxima* (Db1#, Db2#, Db3#, Db4#, and Myxc2#), four of *C. moschata* (Rbf#, Jf8#, Rf9#, and Nf8#), and one variety of *C. andreana* (Xn1#). All varieties were cultivated in Inner Mongolia, except for Xn1#, which was collected from Liaoning province of China. Fresh, fully ripe SUPs were harvested at September 2017. Besides, one variety of pumpkin (Hjg#, *C. pepo*) was used for the comparative study. All the materials were stored in a refrigerator at 4°C until further use.

### 2.2 Chemicals

All solvents and chemicals listed below were analytical grade and purchased from Sinopharm Chemical Reagent Beijing Co., Ltd (Beijing, China): anhydrous ethanol (C_2_H_5_OH), sulfuric acid (H_2_SO_4_), carbazole (C_12_H_9_N), sodium hydroxide (NaOH), ɑ‐naphthol (C_10_H_8_O), oxalic acid (C_2_H_2_O_4_), sodium bicarbonate (NaHCO_3_), sodium chloride (NaCl), n‐hexane (CH_3_(CH_2_)_4_CH_3_), acetone (C_3_H_6_O), BHT, folin‐phenol, sodium carbonate (Na_2_CO_3_), methanol (CH_3_OH), sodium nitrite (NaNO_2_), aluminum hydrate nitrate (Al(NO_3_)_3_.9H_2_O), fructose (C_6_H_12_O_6_), sucrose (C_12_H_22_O_11_), hydrochloric acid (HCl), phenol (C_6_H_5_OH), methyl red (C_15_H_15_N_3_O_2_), zinc acetate (Zn(CH_3_COO)_2_), potassium ferrocyanide (K_4_Fe(CN)_6_.3H_2_O), potassium sodium tartrate (C_4_H_4_O_6_KNa·4H_2_O), 3,5‐dinitrosalicylic acid (C_7_H_4_N_2_O_7_).

Standards of galacturonic acid, rutin, gallic acid, L(＋)‐ascorbic acid, glucose, sucrose, and fructose (purity﹥99%) were of HPLC grade and obtained from the Sigma‐Aldrich (St. Louis, MO, USA.).

### 2.3 By‐products preparation of SUP

SUPs were rinsed by running water, and the seeds were separated from the pulp. The by‐products were cut into slices with 5 mm thickness, which were then frozen in the liquid nitrogen and crushed into a fine powder with a high‐speed grinder (JYL‐C93T, Joyoung, Beijing, China), samples were kept at −20°C prior to chemical analysis.

### 2.4 Determination of physio‐chemical and nutritional properties

#### 2.4.1 Color parameters

Color parameters of different samples were determined by colorimeter (D25L, Hunterlab, Virginia, USA) which was first calibrated with a blackboard and a whiteboard. “L*”, “a*”, and “b*” values which presented lightness, redness/ greenness, yellowness/ blueness, respectively, were used for evaluation of color parameters of different samples. The measurement was repeated 5 times for each sample, and the average value was calculated (Aydin & Gocmen, 2015; Bi et al., 2014).

#### 2.4.2 Soluble solid content

Soluble solid content was tested with a digital display refractometer (WZB 45, Shanghai precision and scientific instrument Co., Ltd.), which should be calibrated with distilled water first until the data showed 0 ºBx. About 10 g SUPB was appropriately crushed in a mortar and then poured onto the gauze with the juice extruded manually. Two drops of the extruded juice were dropped onto the detector, and the value was recorded. The results were expressed as ºBx.

#### 2.4.3 Total phenolic content (TPC) and total flavonoid content (TFC)

*Extraction*: Methanol–water solution was applied for sample extraction which followed the method of Song et al. (2018) Briefly, 3 g of each sample was mixed with 50 ml methanol–water solution (80:20, v: v) and then extracted by ultrasonic (40 kHz, 100 W) for 1 hr at ambient temperature, followed by centrifuging at 3,000*g* for 15 min. Afterward, the supernatant was collected, and the residue was extracted twice according to procedure described above, all the collected supernatant mixing together and then filtered pass through a 0.45 μm microporous membrane. The extraction was stored at −20°C until analysis. UV‐visible spectrophotometer (UV1800, Shimadzu Co. Ltd.) was used for determining the absorbance.

*Determination of TPC*: The TPC was determined according to the procedure of Nistor et al., (2017) with some modifications. Accurate 0.5 ml extract, 2.5 ml of folin‐phenol reagent (100 g/kg), and 2 ml of sodium carbonate (75 g/kg) were added into a tube; then, the mixture was placed at 50ºC for 15 min. Afterward, the absorbance was read at 760 nm against methanol–water solution (80:20, v: v) as a blank. The results were expressed as mg/ 100 g wet basis (w. b.) of gallic acid equivalents (GAE).

*Determination of TFC*: The TFC was determined following the method of Song et al. (2018) with some modifications. Exact 1 ml extract and 0.3 ml NaNO_2_ (50 g/kg) were mixed in a tube, followed by the addition of 0.3 ml Al(NO_3_)_3_ (100 g/kg) and 4 ml NaOH (50 g/kg). After 15 min, the absorbance was read at 510 nm against methanol–water solution (80:20, v: v) as a blank. The TFC of the samples was expressed as mg/100 g w. b. of rutin equivalents (RE).

#### 2.4.4 Pectin content

*Pretreatment and extraction*: Accurate 4 g of SUPs by‐products, 35 ml of the heated anhydrous ethanol (about 75°C) and scraps of paper were added into a tube, which was held in an 85°C water bath (DK‐525, Jinghong experimental equipment Co., Ltd., Shanghai, China) for 10 min. After cooling, another 15 ml heated anhydrous ethanol was added. The extract was then centrifuged at 3,000*g* for 15 min and the supernatant was removed. The residue was submitted to re‐extracted four times with 50 ml heated anhydrous ethanol under the same conditions. All the residues were collected for further determination.

The precipitates prepared above were washed into triangular flasks with pH = 0.5 sulfuric acid solution (0.16 mol/L), followed by 1 min mixing (600 rpm) with a magnetic stirrer (EMSA240167, Ingle technology Co. Ltd., Nanjing, China); then, the mixture was placed in an 85°C shaking water bath (HZS‐HA, Donglian electronic technology development Co. Ltd., Ha'erbin, China) at 100 rpm for 1 hr and diluted to 100 ml with pH = 0.5 sulfuric acid solution (0.16 mol/L). Afterward, the mixture was filtered by a vacuum pump (SHZ‐D (Ⅲ), Yuhua Instruments Co. Ltd., Henan, China) and the supernatant was collected, stored at 4°C until further analysis (NY/T2016‐2011, 2011).

*Determination*: The determination of pectin was according to the classic method described by Nelly and Gustav (Nelly & Gustav, 1973). The results were expressed as mg/ 100 g w. b. of galacturonic equivalents (GE).

#### 2.4.5 Ascorbic acid content (AAC)

The AAC was determined by a classical titration method using 2, 6‐dichlorophenol indophenol solution (Zhong et al., 2017). The results were expressed as mg/ 100 g w. b. of L (＋)‐ascorbic acid equivalents (LAAE).

#### 2.4.6 Total carotenoid content (TCC)

Extraction: SUPBs (3 g) of each variety were added to a vessel which covered by aluminum foil. Exact 50 ml of extraction solvent (n‐hexane/ acetone/ ethanol = 2:1:1, v:v:v, BHT 0.1%, w/ w) was added to the vessel, which was magnetically stirred for 40 min, following by addition of 15 ml distilled water. The upper layer was collected and evaporated to dryness, and the residue was dissolved with n‐hexane to a final volume of 10 ml (Knockaert, Lemmens, Van Buggenhout, Hendrickx, & Van Loey, 2012).

Determination: TCC was measured by a portable UV‐visible spectrophotometer (UV1800, Shimadzu Co. Ltd., Kyoto, Japan) at 450 nm against n‐hexane as a blank. The results were calculated by the concentration of*β*‐carotene in hexane and expressed as mg/ 100 g w. b.

#### 2.4.7 Total sugar content (TSC)and polysaccharides content

*Extraction of total sugar*: 1 g of each sample, 2 ml 6 mol/L HCl, and 20 ml distilled water were added to a tube, which was placed in a 96°C water bath for 2 hr, then cooled immediately using the running water. Accurate 2 ml 6 mol/L NaOH was then added, followed by 15 min centrifuging (3,500*g*, 4°C) with a centrifuge (3K15, sigma, Germany). The supernatant was preserved and set to 50 ml.

*Extraction of polysaccharide*: Each sample was extracted with hot water (1:30, w: v) at 90°C for 1 hr, followed by ultrasonic assisted extraction (40 kHz, 100 W) at ambient temperature for 1 hr. Subsequently, the aqueous extract was concentrated to 10 ml by rotary vacuum evaporator (RE‐3000, Yirong biochemical instrument factory, Shanghai, China) at 30°C. Addition 160 ml anhydrous ethanol was then added, and the mixture was stored at 4°C. After 24 hr, the suspension was collected and diluted to 100 ml with distilled water.

*Determination*: Both TSC and polysaccharide content were determined by the phenol sulfuric acid method described by Wu, Xu, Chi, & Wu (2007) and the results were expressed as g/ 100 g w. b. of glucose equivalents (GE).

#### 2.4.8 Reducing sugar content (RSC) and Soluble sugar content (SSC)

*Extraction*: The extraction of reducing sugar and soluble sugar was both according to the method reported by Si, Dai, Tian, Sun, & Wang (2001).

*Determination of RSC*: Exact 0.4 ml of the extract was placed in a 10 ml test tube, mixed with 1.6 ml distilled water and 4 ml 3,5‐dinitrosalicylic acid, which was kept in boiled water for 5 min. Another 4 ml distilled water was then added after the sample was cooled to ambient temperature. The RSC in the samples was measured by the UV‐visible spectrophotometer (UV1800, Shimadzu Co. Ltd., Kyoto, Japan) at 540 nm against distilled water as a blank. The results were expressed as g/ 100g w. b of glucose equivalents (GE).

*Determination of SSC*: Accurate 10 ml of the extract was reacted with 1 ml 6 mol/L HCl, which heated in an 85°C water bath for 10 min. After cooling, the mixture was neutralized to a light orange by 6 mol/L NaOH solution with methyl red as the indicator, up to a final volume of 100 ml with distilled water. Two milliliters of the solution and 4 ml 3,5‐dinitrosalicylic acid were mixed together. The following procedure was as the same as the determination of reducing sugar. The SSC was expressed as g/ 100 g w. b of glucose equivalents (GE).

#### 2.4.9 Glucose, sucrose, and fructose contents

*Extraction*: A sample of 0.5 g was added to 50 ml distilled water in a tube and then extracted by ultrasonic (40 kHz, 100 W) for 1 hr at ambient temperature. The supernatant was collected and filtered through 0.45 µm filter, stored at 4°C until analysis.

*Determination*: Glucose, sucrose, and fructose content were determined by the high‐performance anion‐exchange chromatography with pulsed amperometric detection (HPAEC‐PAD) described by Song et al. (2019) and the extract used in this study was 10 µl. The contents of glucose, sucrose, and fructose were expressed as g/ 100 g w. b.

#### 2.4.10 Other indices

Water content, crude protein content (CPC), crude fiber content, and crude fat content (CFC) were determined according to the National standards of the People's Republic of China (GB5009.3‐2016, 2016; GB5009.5‐2016, 2016; GB5009.6‐2016, 2016; GB/T5009.10‐2003, 2003). Mineral elements were measured by the method described by Oliveira et al. (2014) The contents of these indices were expressed as g/ 100 g w. b.

### 2.5 Aroma profile determination by E‐nose

A commercial PEN 3.5 electronic nose (E‐nose, Airsense Analytics, GmBH, Schwerin, Germany) was used, which had ten gas sensors that were sensitive to different types of aroma compounds. The operating method was described by Chen, Song, Bi, & Meng (2018) Briefly, exact 2 g sample was placed in an airtight glass vial, which was closely capped with a PTFE‐silicon stopper, and the headspace gaseous compounds were pumped into the sensor arrays through Teflon tubing connected to a needle in the plastic. The respond value was recorded every second and expressed as G/ G0 (G and G0 represented sample gas and zero gas, respectively). Each measurement was carried out for 60 s and repeated in triplicate.

### 2.6 Statistical analysis

All the experiments were at least conducted in triplicates and the mean values ± standard deviations were recorded. Analysis of variance (ANOVA) and Duncan's test were used to determine the significance of difference among different varieties. *PCA* and *SCA* of different indices were analyzed by the software of SPSS22.0 (IBM, Chicago, USA). The color map of values and correlation analysis of 27 indices were obtained by HemI 1.0 (http://ccd.biocuckoo.org/). *PCA* and *LDA* of E‐nose data were performed using the software of Winmuster. EZinfo 3.0 software (Umetrics AB, Umea, Sweden) was used for *PCA‐X* model analysis.

## 3 RESULTS AND DISCUSSION

### 3.1 Physico‐chemical and nutritional quality analysis of SUP by‐products

#### 3.1.1 Characterization of 27 evaluation indices of SUP by‐products

**Figure 1:**
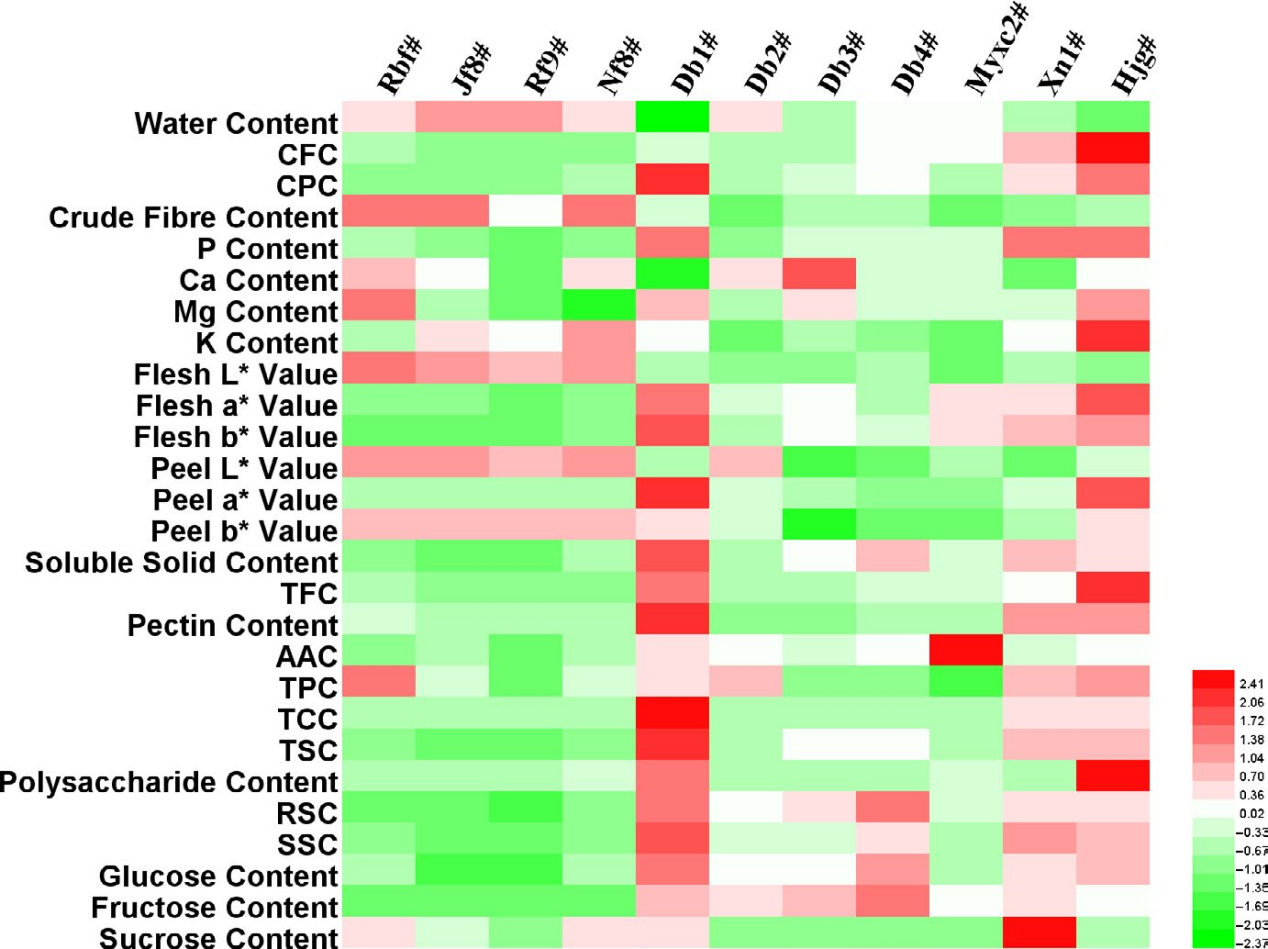
The color map of physico‐chemical and nutritional contents of SUP by‐products. Note: Color from green to white to red means contents of different indices varied from lowest to medium to highest

The coefficient of variation (CV) of 27 indices of different SUP by‐products were shown in Table 1. CV values were in the range of 4.57% ~ 200.83%. Water content showed the lowest CV value (4.57%), and the minimum water content was presented in Db1# (81.84 g/ 100 g, Table S1). CFC presented significant differences (*p* < .05) among different varieties which ranging from 0.07 (Rf9# and Jf8#) to 0.35 g/ 100 g (pumpkin, Hjg#). Meanwhile, Db1# was characterized by the highest CPC value (2.28 g/ 100 g, Table S1) in the by‐products of SUP. In terms of mineral elements, phosphorus (P) showed the highest CV value of 77.19% which contents were in the range of 4.39 ~ 42.53 mg/ 100 g (Table S1). Moreover, “P” in Xn1# and Db1# were comparable with that of Hjg# (Figure 1). Potassium (K) was the most abundant element in by‐products of SUP but showed the lowest CV value (33.95%) among mineral element. The variation of “K” was from 108.41 (Myxc2#) to 349.79 (Nf8#) mg/ 100 g. Highest contents of “Mg” and “Ca” were found in by‐products of Rbf# (21.64 mg/ 100 g) and Db3# (80.16 mg/ 100 g), respectively.

**Table 1:** Variation of 27 indices for the by‐products of seed‐used pumpkins

Quality parameter	Mean	*SD*	Minimum	Maximum	Coefficient of variation (%)	Significance
Water Content ( g/ 100 g)	92.59	4.23	81.84	96.92	4.57	**
Crude Fat Content ( g/ 100 g)	0.15	0.09	0.07	0.35	60.00	**
Crude Protein Content ( g/ 100 g)	0.83	0.56	0.32	2.28	67.47	**
Crude Fiber Content ( g /100 g)	1.37	0.54	0.77	2.16	39.42	**
P Content ( mg/ 100 g)	17.23	13.30	4.39	42.53	77.19	**
Ca Content ( mg/ 100 g)	51.78	15.32	26.30	80.16	29.59	**
Mg Content ( mg/ 100 g)	13.81	4.16	6.34	21.64	30.12	**
K Content ( mg/ 100 g)	220.51	74.87	108.41	349.79	33.95	**
Flesh L* Value	63.64	13.41	48.40	80.90	21.07	**
Flesh a*Value	8.65	8.41	−0.40	25.87	97.23	**
Flesh b* Value	38.90	11.87	25.96	63.30	30.51	**
Peel L* Value	60.94	18.44	32.91	80.36	30.26	**
Peel a *Value	6.00	12.05	−2.57	40.46	200.83	**
Peel b* Value	26.07	13.38	4.60	39.71	51.32	**
Soluble Solid Content ( ºBx)	5.30	3.19	1.70	11.78	60.19	**
Total Flavonoid Content ( mg/ 100 g)	11.25	5.91	5.17	26.08	52.53	**
Pectin Content ( mg/ 100 g)	516.64	280.21	305.01	1,166.15	54.24	**
Ascorbic Acid Content ( mg/ 100 g)	1.38	0.94	0.45	3.81	68.12	**
Total Phenolic Content ( mg/ 100 g)	22.18	7.68	12.33	35.50	34.63	**
Total Carotenoid Content ( mg/ 100 g)	3.07	6.00	0.20	19.57	195.44	**
Total Sugar Content ( g/ 100 g)	4.90	3.77	1.13	13.69	76.94	**
Polysaccharide Content ( g/ 100 g)	0.46	0.46	0.22	1.80	100.00	**
Reducing Sugar Content ( g/ 100 g)	2.55	1.77	0.24	5.24	69.41	**
Soluble Sugar Content ( g/ 100 g)	3.58	2.22	1.09	7.95	62.01	**
Glucose Content ( g/ 100 g)	1.17	0.66	0.15	2.21	56.41	**
Fructose Content ( g/ 100 g)	1.32	1.07	0.02	2.92	81.06	**
Sucrose Content ( g/ 100 g)	0.54	0.70	0.00	2.20	129.63	**

** *p* < .01

“L*”, “a*”, and “b*” values of peel and flesh of SUP by‐products were evaluated. As shown in Table 1, “a*” value of peel and flesh showed higher CV values (200.83% and 97.23%, respectively) than “L*” and “b*”. Varieties of Rbf#, Jf8#, and Nf8# which were in family of *C. moschata* had no significant differences in “a*” value of peel. Db1# had the highest “a*” value both in peel and flesh, which the red‐yellow color was the closest to Hjg#, whereas Db3#, Db4#, Myxc2#, and Xn1# showed minus peel “a*” value, which was consistent with the green appearance which shown in Figure S1. However, flesh “a*” value of *C. maxima* (Db1#, Db2#, Db3#, Db4#, Myxc2#) was significantly higher than *C. moschata* (Rbf#, Jf8#, Rf9#, Nf8#). The similarity and differences of the results could be explained by the different varieties and planting conditions.

The CV values of nutritional indices of SUP by‐products were significantly affected by different varieties from 34.63% (TPC) to 195.44% (TCC). Among all the varieties, Db1# had the highest value of soluble solid content (11.78 ºBx), pectin content (1,166.15 mg/100 g), TCC (19.57 mg/100 g), TSC (13.69 g/100 g), RSC (5.24 g/ 100 g), SSC (7.95 g/100 g), and glucose content (2.21 g/100 g), which were even higher than those of Hjg# (Table S2). TCC showed the highest CV (195.44%) which was varied from 0.20 to 19.57 mg/100 g; however, there was no significant difference among TCC values of varieties in Rbf#, Jf8#, Rf9#, Nf8#, Db2#, and Db4#. Polysaccharide and sucrose contents had the CV values that over 100%. Db1# had the highest polysaccharide content (Figure 1) among by‐products of SUP, but still lower than Hjg# (2.75 g/ 100 g). The highest sucrose content was found in Xn1# (2.2 g/ 100 g), which was significantly higher than Hjg#. According to Figure 1, a relatively lower contents of total sugar, reducing sugar, soluble sugar, and fructose were found in the samples of Rbf#, Jf8#, Rf9#, and Nf8#, but a relatively higher sucrose content was presented in them, except for Rf9#.

#### 3.1.2 Correlation analysis (CA)

**Figure 2:**
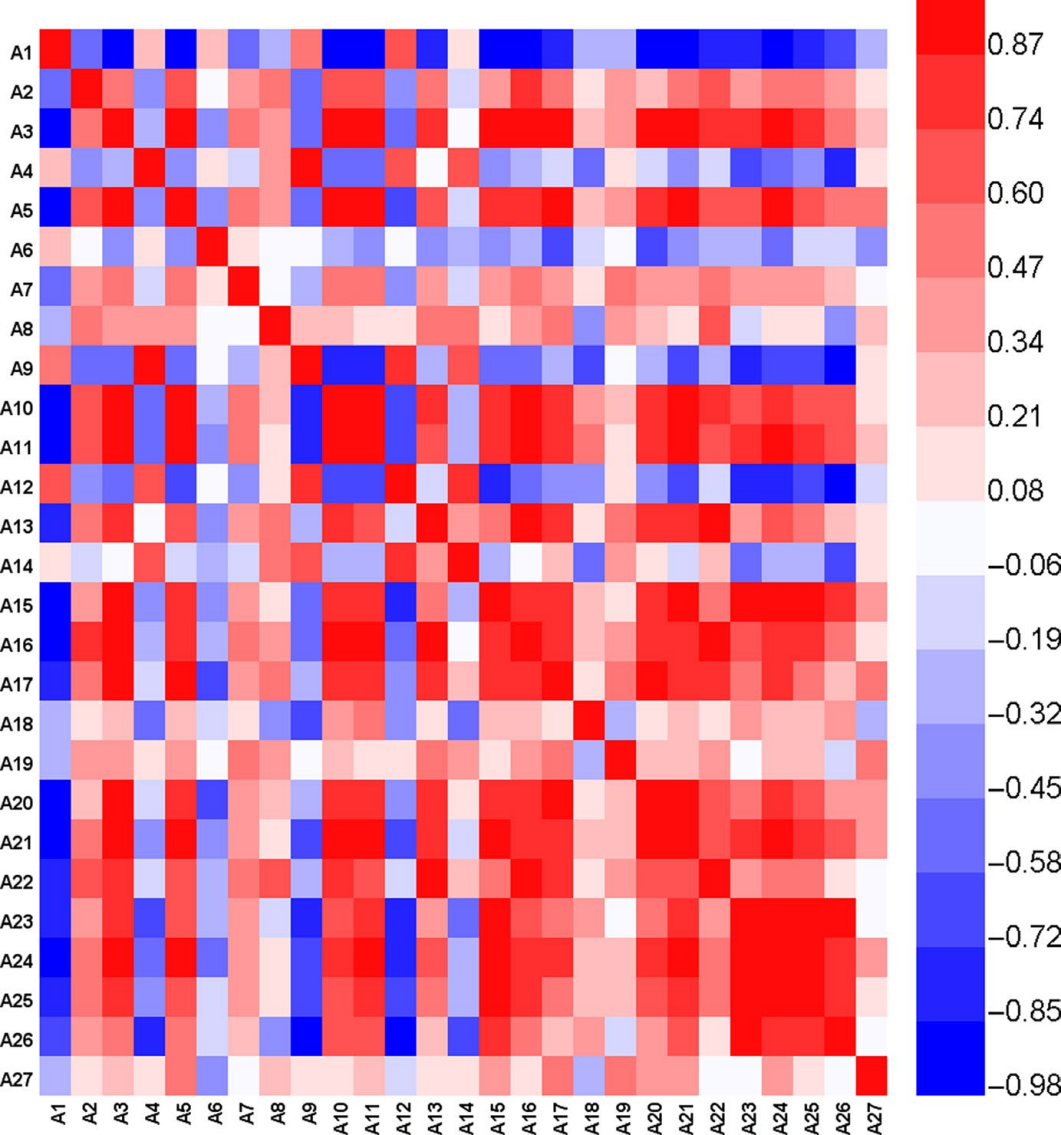
Correlation analysis on 27 evaluation indices. A1‐A27: Water Content, Crude Fat Content, Crude Fiber Content, Crude Protein Content, Soluble Solid Content, P Content, Ca Content, Mg Content, K Content, Total Flavonoid Content, Pectin Content, AA Content, Total Phenolic Content, Total Carotenoid Content, Total sugar content, Polysaccharide Content, Reducing Sugar Content, Soluble Sugar Content, Glucose Content, Fructose Content, Sucrose Content, Flesh “L*” Value, Flesh “a*” Value, Flesh “b*” Value, Peel “L*” Value, Peel “a*” Value, Peel “b*” Value. Color from blue to white to red means the correlation coefficient values from lowest to highest (−1 to 1)

The correlation analysis of physio‐chemical and nutritional indices was conducted, and the results were presented in Figure 2. A1 to A27 represented water content, CFC, crude fiber content, CPC, soluble solid content, P content, Ca content, Mg content, K content, TFC, pectin content, AA content, TPC, TCC, TSC, polysaccharide content, RSC, SSC, glucose content, fructose content, sucrose content, flesh “L*” value, flesh “a*” value, flesh “b*” value, peel “L*” value, peel “a*” value, peel “b*” value, respectively. Exact 240 pairs of positive correlations and 116 pairs of negative correlations were found between different indices at *p* < .05 (Table S3). According to Figure 2, the water content was significantly negative with most indices (*p* < .05); however, it showed significantly positive relationship with flesh and peel “L*” value. Apart from sucrose, all the other sugars were negatively related to flesh “L*” value and peel “L*” value but presented significantly positive correlations with flesh “a*” value and flesh “b*” value (*p* < .01). TCC was a color‐forming substance itself and had a certain effect on the color of samples (Meléndez‐Martínez, Britton, Vicario, & Heredia, 2006); hence, it was significantly positively correlated with flesh “a*” value, flesh “b*” value and peel “a*” value (*p* < .01). Due to the close relationship between metabolic pathways of carotenoid and sugars (Yu, Weng, & Zhou, 2011), TCC was also significantly positively related to TSC, SSC (*p* < .01), polysaccharide content, and glucose content (*p* < .05).

#### 3.1.3 Principal components analysis (PCA)

In order to evaluate the comprehensive quality of the by‐products of SUPs accurately and quickly, several principal components which provide more scientific and effective data information could be obtained through *PCA *(Syms, 2008; Wold, Esbensen, & Geladi, 1987). In this study, the first five principle components (PCs) accounted for 42.7%, 25.4%, 9.7%, 7.0%, and 6.6%, respectively, with cumulative contribution of 91.4% (> 90%), which were quite enough to explain the total data variability (Chen et al., 2018; Hong, Wang, & Qi, 2015). As presented in Table S4, PC_1_ showed high correlation with five indices: TCC (+), peel “a*” value (+), CPC (+), pectin content (+), and water content (‐). PC_2_ mainly correlated with peel “b*” value (‐), flesh “L*” value (‐), peel “L*” value (‐), and fructose content (+). PC_3_, PC_4,_ and PC_5_ were highly contributed by CFC (CV = 60.00%), sucrose content (CV = 129.63%), and Ca content, respectively. For discriminate the representative indices in PC_1_ and PC_2_, *PCA‐X* model analysis was further used.

#### 3.1.4 PCA‐X model analysis

**Figure 3:**
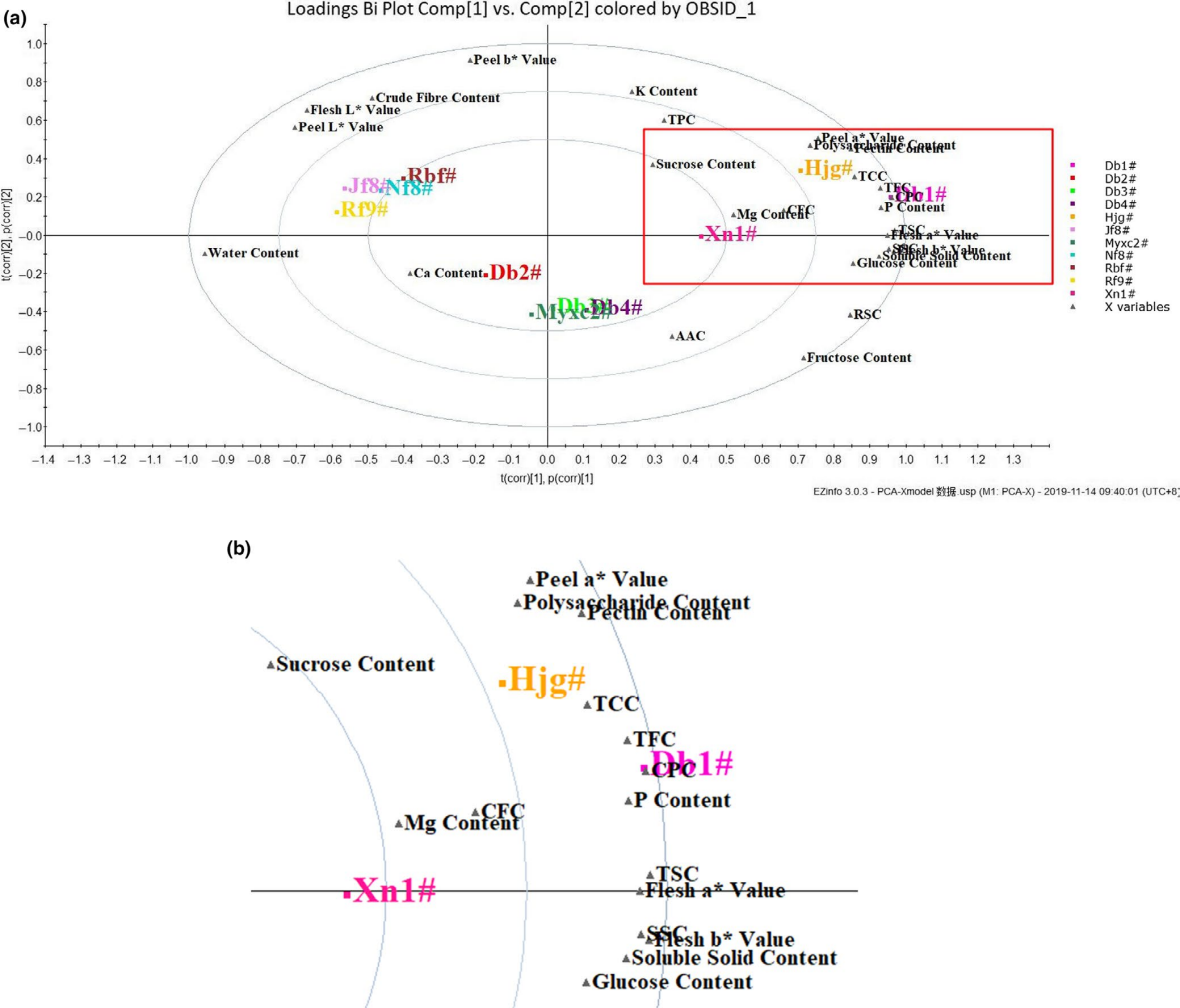
PCA‐X model analysis on 27 evaluation indices and different samples. Note: (b) was the contents of the red box in (a)

According to Figure 3 (a), the plot of water content was far from other indices, which indicated that it had significant effect on the quality of SUPB. Besides, water was the most abundant component in SUPBs (81.84 ~ 96.92 g /100 g); moreover, it significantly negatively related to most indices (*p* < .01, Table S3). Hence, water content could be considered one representative indicator in PC_1_. The plots of peel “a*” value, CPC, pectin content, and TCC were closed to each other, proving they had overlapped information. Peel “a*” value had the highest CV (200.83%), and significantly positively related to CPC, pectin content, and TCC (*p* < .01, Table S3), which made it the other representative indicator in PC_1_. In terms of PC_2_, the plot of fructose was far from the other three indices, which also had a relatively higher CV (81.06%). Six characteristic indicators were finally screened out through CV, *CA*, *PCA,* and *PCA‐X* model: peel “a*” value, water content, fructose content, CFC, sucrose content, and Ca content.

#### 3.1.5 System cluster analysis (SCA)

Cluster analysis is a statistical analysis technique that divides a group of subjects into groups of relative homogeneity (Zhuang & Gao, 2018). In this research, *SCA* was an essential way to screen out some SUPs varieties with high nutritional quality.

**Figure 4:**
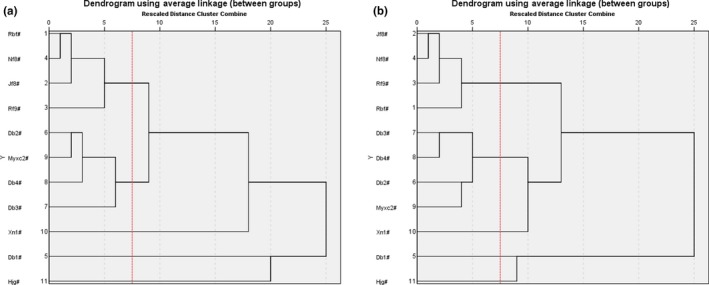
Dendrogram of system cluster analysis for different samples based on 6 characteristic indicators (a) and 27 indices (b)

According to the 6 characteristic indicators mentioned above, the samples were grouped into five clusters at 7.5 Euclidean distance. As presented in Figure 4(a), Hjg# was clearly separated from SUPs, which accommodated into one Cluster. Among 10 varieties of SUPs, Jf8#, Nf8#, Rbf#, and Rf9# which in the family of *C. moschata*, they presented similar appearance (Figure S1) and the values of most indices (Tables S1 and S2); thus, they were grouped into Cluster I. Db4#, Myxc2#, Db2#, and Db3# belonged to *C. maxima*, with similar values of TFC and sucrose content, accommodated into Cluster II. Although as one variety of *C. maxima*, Db1# had a relatively higher nutritional value, with the highest values of soluble solid content (11.78 ºBx), pectin content (1,166.15 mg/ 100 g), TCC (19.57 mg/ 100 g), TSC (13.69 g/ 100 g), RSC (5.24 g/ 100 g), SSC (7.95 g/ 100 g), and glucose content (2.21 g/ 100 g, Table S2), which accommodated into Cluster Ⅲ. Only Xn1# belonged to the species of *C. andreana*, which showed unique quality performance and mainly used for grafting in the industry, accommodated into Cluster Ⅳ. Meanwhile, the same cluster results could also be proved by *PCA‐X* model analysis (Figure 3). To validate the accuracy of results that analyzed by 6 selecting characteristic indicators, *SCA* analysis based on 27 indices had also been done, and the same result was obtained (Figure 4b). Hence, physio‐chemical and nutritional quality evaluation of SUPB could be done by determining indices of peel “a*” value, water content, fructose content, CFC, sucrose content, and Ca content.

### 3.2 Volatile profile analysis of SUP by‐products

#### 3.2.1 E‐nose determination

**Figure 5:**
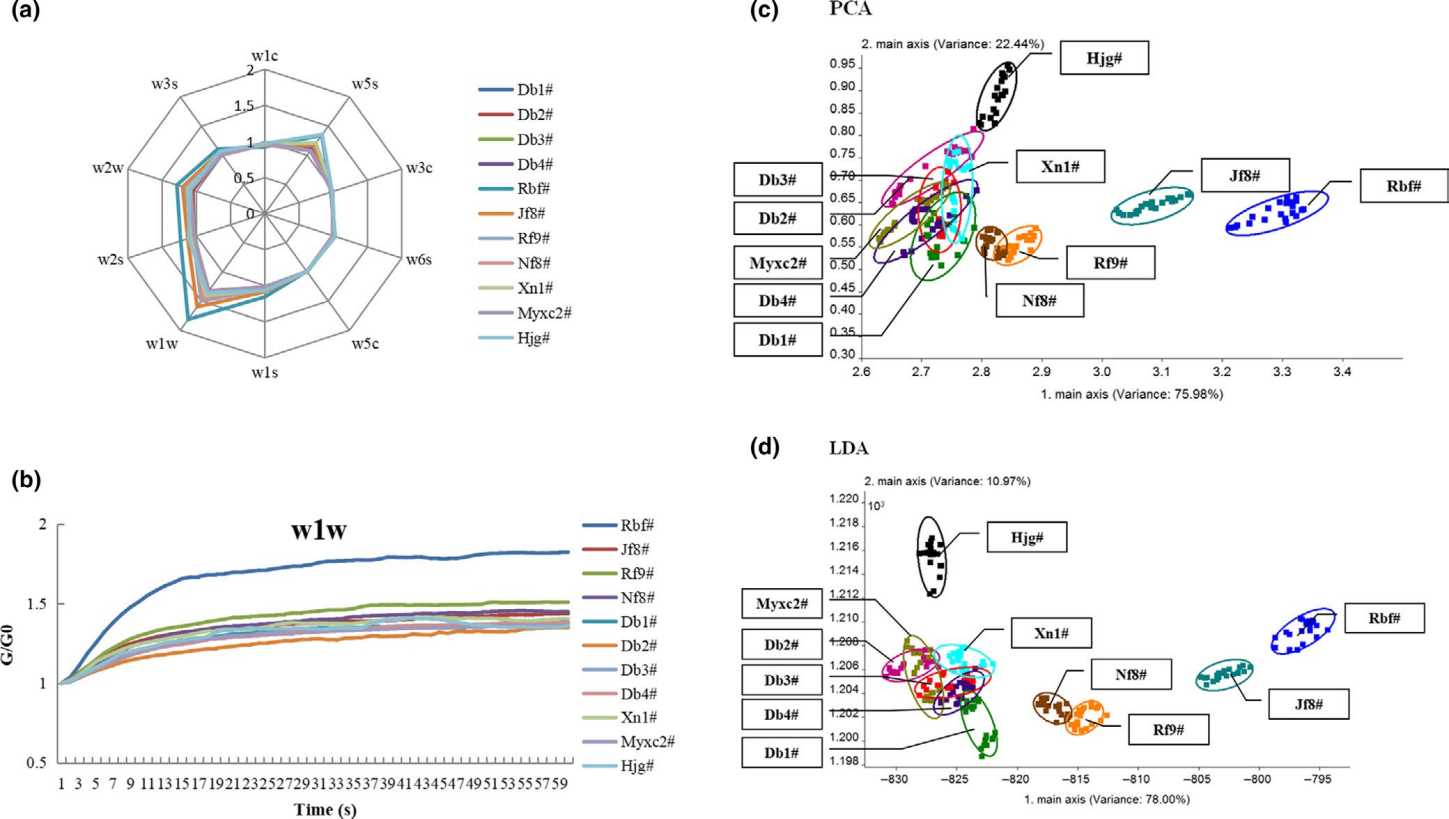
E‐nose data of different by‐products of seed‐used pumpkins. (a): Radar chat of E‐nose responses, (b): Respond values of W1W, (c): LDA by E‐nose, (d): PCA by E‐nose

Aroma was another important attribute of fruit quality, and the E‐nose was applied to distinguish the aroma difference of different varieties. In this study, the response values (G/ G0) of 11 samples to ten different sensors were shown in radar chat of Figure 5a. There were significant differences in the response values of W1W (sensitive to terpenes and sulfur‐containing organic compound) and W5S (broadrange) among different varieties. Typical E‐nose responses (W1W) were presented in Figure 5b, where response values (G/ G0) gradually increased and finally reached a stable level. W1W was more sensitive to the by‐products of Rbf#, which indicated that more aromatic components were produced in Rbf# (such as terpenes, methane, and organic sulfides). However, different by‐products of SUP samples could not be well distinguished just by the sensor signals. So, *LDA* and *PCA* were further used in this work.

#### 3.2.2 LDA and PCA on volatile profile of different varieties

*LDA* and *PCA* results based on E‐nose data were given in Figure 5c,d. The accumulative contribution of LD_1_ and LD_2_, PC_1_ and PC_2_ were 88.97% and 98.42%, respectively, which were sufficient enough to express the total variance of 11 samples (Hong et al., 2015). According to the results of *LDA* and *PCA*, Hjg# was clearly separated from SUPs. Rbf#, Jf8#, Nf8#, and Rf9# could be discriminated from the other varieties, which could be classified as Cluster Ⅰ, while Db1#, Db2#, Db3#, Db4#, Myxc2#, and Xn1# were classified as Cluster Ⅱ.

In most cases, cluster analysis results based on aroma profile showed agreement with that of based on physio‐chemical and nutritional indices. However, there were some differences between them. Both Db1# and Xn1# presented similar aroma profile with Db2#, Db3#, Db4#, Myxc2#; however, significant differences were shown based on analysis of physico‐chemical and nutritional indices. Hence, comprehensive analysis was essential to quality evaluation of SUPB. In total, Db1# showed higher nutritional value and favorable aroma could be a good food resource for deep processing.

## 4 CONCLUSIONS

Physico‐chemical, nutritional, and aroma profile of SUPBs were investigated. Six characteristic evaluation indicators of SUPBs (peel “a*” value, water content, fructose content, CFC, sucrose content, and Ca content) were screened by *CA*, *PCA,* and *PCA‐X* model analysis. Among all the varieties of SUPB, Db1# was showed higher nutritional value and favorable aroma could be a good food resource for deep processing.

## CONFLICT OF INTEREST

All the authors declare that they have no conflict of interest.

## ETHICAL APPROVAL

This study does not involve any human or animal testing.
