# Supplementary material for: Quality assessment and variety classification of seed‐used pumpkin by‐products: Potential values to deep processing
Source: Food Sci Nutr. 2019 Nov 19;7(12):4095–104. doi: 10.1002/fsn3.1276 (PMC6924301; doi:10.1002/fsn3.1276)
Supplement: Supplementary file 2 [file FSN3-7-4095-s002.docx]

**Table S1. The physico-chemical indices of SUPBs**

| Samples | Cultivar | Water  （g/100g） | Crude Fat（g/100g） | Crude Protein（g/100g） | Crude Fibre（g/100g） | P  （mg/100g） | Ca  （mg/100g） | Mg  （mg/100g） | K  （mg/100g） | Flesh L | Flesh a | Flesh b | Peel L | Peel a | Peel b |
| --- | --- | --- | --- | --- | --- | --- | --- | --- | --- | --- | --- | --- | --- | --- | --- |
| Seed-used Pumpkin | Rbf# | 94.99±0.02 ^c^ | 0.10±0.00 ^efg^ | 0.50±0.00 ^i^ | 2.10±0.04 ^a^ | 9.72±0.00 ^d^ | 64.60±0.03 ^b^ | 21.64±0.09 ^a^ | 199.65±0.12 ^g^ | 80.90±0.07 ^a^ | 0.64±0.08 ^h^ | 27.44±0.14 ^h^ | 80.36±0.31^a^ | 2.34±0.28^d^ | 37.63±0.23^b^ |
|  | Jf8# | 96.67±0.04 ^b^ | 0.07±0.05 ^g^ | 0.38±0.02 ^j^ | 2.16±0.26 ^a^ | 9.60±0.02 ^d^ | 53.20±0.10 ^f^ | 11.99±0.02 ^h^ | 296.23±0.11 ^c^ | 80.76±0.08 ^a^ | 1.02±0.38 ^h^ | 25.96±0.15 ^h^ | 79.36±0.11^ab^ | 3.14±0.29^d^ | 38.34±0.31^a^ |
|  | Rf9# | 96.92±0.02 ^a^ | 0.07±0.00 ^g^ | 0.32±0.02 ^k^ | 1.38±0.11 ^b^ | 4.39±0.00 ^g^ | 36.81±0.12 ^i^ | 9.50±0.04 ^i^ | 249.94±0.38 ^e^ | 76.25±0.10 ^b^ | -0.40±0.08 ^i^ | 27.43±0.09 ^h^ | 78.33±0.23^b^ | 3.76±0.17^d^ | 39.71±0.21^a^ |
|  | Nf8# | 94.29±0.00 ^e^ | 0.08±0.02 ^fg^ | 0.59±0.02 ^g^ | 2.09±0.01 ^a^ | 6.86±0.03 ^e^ | 62.15±0.13 ^c^ | 6.34±0.18 ^j^ | 349.79±0.16 ^b^ | 79.57±0.08 ^a^ | 0.90±0.13 ^h^ | 29.58±0.30 ^g^ | 79.7±0.20^ab^ | 2.73±0.10^d^ | 39.10±0.19^a^ |
|  | Db1# | 81.84±0.00 ^k^ | 0.15±0.00 ^d^ | 2.28±0.01 ^a^ | 1.34±0.04 ^bc^ | 40.77±0.01 ^b^ | 26.30±0.21 ^j^ | 17.73±0.32 ^c^ | 246.85±0.06 ^f^ | 56.75±0.31 ^c^ | 25.87±0.59 ^b^ | 63.30±0.49 ^a^ | 48.63±2.07^e^ | 40.46±2.20^a^ | 35.66±0.11^b^ |
|  | Db2# | 94.33±0.00 ^d^ | 0.12±0.01 ^def^ | 0.53±0.01 ^h^ | 0.78±0.11 ^e^ | 5.86±0.03 ^f^ | 59.67±0.09 ^d^ | 12.52±0.12 ^g^ | 117.02±0.12 ^j^ | 50.84±0.87 ^f^ | 8.68±0.61 ^f^ | 36.13±0.20 ^f^ | 73.25±1.50^c^ | 7.66±1.33^c^ | 26.29±2.56^c^ |
|  | Db3# | 90.55±0.01 ^h^ | 0.14±0.01 ^de^ | 0.93±0.00 ^e^ | 1.01±0.08 ^d^ | 17.47±0.00 ^c^ | 80.16±0.64 ^a^ | 17.03±0.13 ^d^ | 192.00±0.08 ^h^ | 50.37±1.15 ^f^ | 12.07±0.38 ^e^ | 44.12±0.53 ^e^ | 32.91±1.26^h^ | -0.51±0.51^e^ | 4.60±1.32^f^ |
|  | Db4# | 92.60±0.01 ^g^ | 0.24±0.00 ^c^ | 0.96±0.02 ^d^ | 1.09±0.03 ^d^ | 17.52 ±0.02 ^c^ | 50.59±0.11 ^g^ | 13.71±0.02 ^f^ | 169.52±0.43 ^i^ | 57.32±1.10 ^c^ | 6.31±0.62 ^g^ | 37.46±0.52 ^f^ | 42.10±0.72^f^ | -2.57±1.29^f^ | 10.15±1.01^e^ |
|  | Myxc2# | 93.48±0.00 ^f^ | 0.21±0.04 ^c^ | 0.63±0.02 ^f^ | 0.77±0.24 ^e^ | 17.60±0.02 ^c^ | 47.88±0.40 ^h^ | 14.43±0.14 ^e^ | 108.41±0.13 ^k^ | 48.40±2.07 ^g^ | 14.41±1.03 ^d^ | 46.43±1.44 ^d^ | 54.40±0.10^d^ | -1.16±1.03^ef^ | 11.86±1.04^e^ |
|  | Xn1# | 90.22±0.00 ^i^ | 0.35±0.01 ^a^ | 1.16±0.01 ^c^ | 0.98±0.01 ^de^ | 42.53±0.52 ^a^ | 36.43±0.07 ^i^ | 13.50±0.06 ^f^ | 275.72±0.09 ^d^ | 55.24±0.18 ^d^ | 17.02±0.86 ^c^ | 51.17±1.94 ^c^ | 40.34±0.41^g^ | 4.19±0.19^d^ | 17.05±2.70^d^ |
| Range |  | 81.84-96..92 | 0.07-0.35 | 0.32-2.28 | 0.77-2.16 | 4.39-42.53 | 26.30-80.16 | 6.34-21.64 | 108.41-349.79 | 48.40-80.90 | -0.40-25.87 | 25.96-63.30 | 32.91-80.36 | -2.57-40.46 | 4.60-39.71 |
| CV. % |  | 4.57 | 60.00 | 67.47 | 39.42 | 77.19 | 29.59 | 30.12 | 33.95 | 21.07 | 97.23 | 30.51 | 30.26 | 200.83 | 51.32 |
| Pumpkin | Hjg# | 87.24±0.00 ^j^ | 0.69±0.04 ^a^ | 1.86±0.01 ^b^ | 1.16±0.00 ^cd^ | 40.77±0.34 ^b^ | 57.15±0.02 ^e^ | 20.14±0.09 ^b^ | 466.38±0.53 ^a^ | 53.36±0.75 ^e^ | 28.07±0.88 ^a^ | 57.21±2.00 ^b^ | 55.05±0.47^d^ | 33.59±1.28^b^ | 35.79±0.48^b^ |

Note:Results are expressed as mean ± SD (n=3). Mean values with different lower-case letters in the same column showed significant differences at *p* < 0.05.

| Samples | Cultivar | Soluble Solid（ºBx） | Total Flavonoid（mg/100g） | Pectin  （mg/100g） | Ascorbic acid（mg/100g） | Total Phenolic（mg/100g） | Total Carotenoid（mg/100g） | Total Sugar（g/100g） | Polysaccharide（g/100g） | Reducing Sugar（g/100g） | Soluble Sugar（g/100g） | Glucose  （g/100g） | Fructose（g/100g） | Sucrose  （g/100g） |
| --- | --- | --- | --- | --- | --- | --- | --- | --- | --- | --- | --- | --- | --- | --- |
| Seed-used Pumpkin | Rbf# | 3.20±0.08 ^f^ | 9.63±0.06 ^f^ | 498.00±5.96 ^c^ | 0.48±0.00 ^e^ | 35.50±1.07 ^a^ | 0.21±0.01 ^de^ | 2.36±0.02 ^h^ | 0.36±0.02 ^d^ | 0.89±0.02 ^h^ | 2.00±0.06 ^i^ | 1.00±0.05 ^e^ | 0.02±0.02 ^h^ | 0.97±0.06 ^b^ |
|  | Jf8# | 2.10±0.25 ^g^ | 5.97±0.37 ^g^ | 438.09±1.08 ^d^ | 0.90±0.11 ^d^ | 21.45±2.36 ^d^ | 0.20±0.02 ^e^ | 1.30±0.08 ^i^ | 0.22±0.00 ^e^ | 0.39±0.01 ^i^ | 1.21±0.06 ^j^ | 0.27±0.05 ^g^ | 0.01±0.01 ^h^ | 0.37±0.02 ^c^ |
|  | Rf9# | 1.70±0.17 ^g^ | 5.17±0.30 ^g^ | 359.01±1.12 ^h^ | 0.45±0.06 ^e^ | 13.76±1.32 ^ef^ | 0.25±0.01 ^de^ | 1.13±0.07 ^j^ | 0.34±0.01 ^d^ | 0.24±0.01 ^j^ | 1.09±0.01 ^j^ | 0.15±0.01 ^h^ | 0.22±0.02 ^g^ | - |
|  | Nf8# | 3.50±0.16 ^f^ | 6.50±0.14 ^g^ | 367.00±5.99 ^g^ | 0.96±0.03 ^d^ | 21.58±0.83 ^d^ | 0.21±0.01 ^de^ | 2.48±0.06 ^h^ | 0.42±0.00 ^c^ | 1.15±0.02 ^g^ | 2.13±0.01 ^h^ | 0.98±0.09 ^e^ | 0.15±0.03 ^g^ | 0.78±0.10 ^b^ |
|  | Db1# | 11.78±0.66 ^a^ | 26.08±2.34 ^b^ | 1166.15±2.33 ^a^ | 1.93±0.32 ^b^ | 26.37±1.55 ^c^ | 19.57±0.94 ^a^ | 13.69±0.07 ^a^ | 1.80±0.06 ^b^ | 5.24±0.01 ^a^ | 7.95±0.15 ^a^ | 2.21±0.05 ^a^ | 2.37±0.16 ^b^ | 0.95±0.03 ^b^ |
|  | Db2# | 3.64±0.24 ^f^ | 10.22±0.24 ^ef^ | 305.01±1.64 ^j^ | 1.61±0.02 ^c^ | 29.42±4.45 ^b^ | 0.24±0.01 ^de^ | 3.76±0.17 ^f^ | 0.22±0.00 ^e^ | 3.18±0.10 ^e^ | 3.21±0.10 ^f^ | 1.31±0.03 ^d^ | 1.85±0.01 ^d^ | 0.03±0.01 ^d^ |
|  | Db3# | 5.56±0.65 ^d^ | 9.51±0.03 ^f^ | 325.03±2.85 ^i^ | 1.13±0.13 ^d^ | 16.10±0.17 ^e^ | 0.99±0.02 ^cd^ | 6.13±0.06 ^d^ | 0.24±0.00 ^e^ | 3.40±0.01 ^d^ | 3.54±0.02 ^e^ | 1.27±0.00 ^d^ | 2.08±0.05 ^c^ | 0.03±0.00 ^d^ |
|  | Db4# | 8.66±0.76 ^b^ | 12.74±1.03 ^d^ | 374.10±1.41 ^f^ | 1.45±0.14 ^c^ | 15.78±0.09 ^e^ | 0.32±0.03 ^de^ | 5.84±0.01 ^e^ | 0.24±0.01 ^e^ | 5.07±0.06 ^b^ | 5.13±0.08 ^d^ | 2.13±0.01 ^a^ | 2.92±0.04 ^a^ | 0.04±0.01 ^d^ |
|  | Myxc2# | 4.50±0.38 ^e^ | 11.41±0.21 ^de^ | 406.05±5.44 ^e^ | 3.81±0.12 ^a^ | 12.33±0.03 ^f^ | 1.18±0.09 ^c^ | 3.49±0.04 ^g^ | 0.45±0.01 ^c^ | 2.50±0.03 ^f^ | 2.90±0.05 ^g^ | 0.86±0.01 ^f^ | 1.60±0.08 ^e^ | 0.02±0.00 ^d^ |
|  | Xn1# | 8.38±0.22 ^b^ | 15.23±1.32 ^c^ | 928.00±3.80 ^b^ | 1.10±0.10 ^d^ | 29.55±1.03 ^b^ | 7.53±0.77 ^b^ | 8.78±0.12 ^b^ | 0.35±0.00 ^d^ | 3.48±0.01 ^c^ | 6.60±0.09 ^b^ | 1.51±0.12 ^c^ | 1.93±0.06 ^d^ | 2.20±0.55 ^a^ |
| Range |  | 1.70-11.78 | 5.17-26.08 | 305.01-1166.15 | 0.45-3.81 | 12.33-35.50 | 0.20-19.57 | 1.13-13.69 | 0.22-1.80 | 0.24-5.24 | 1.09-7.95 | 0.15-2.21 | 0.02-2.92 | 0.00-2.20 |
| CV. % |  | 60.19 | 52.53 | 54.24 | 68.12 | 34.63 | 195.44 | 76.94 | 100.00 | 69.41 | 62.01 | 56.41 | 81.06 | 129.63 |
| Pumpkin | Hjg# | 7.24±0.03 ^c^ | 30.13±1.50 ^a^ | 923.02±6.86 ^b^ | 1.42±0.19 ^c^ | 33.40±1.19 ^a^ | 7.52±0.68 ^b^ | 8.51±0.08 ^c^ | 2.75±0.08 ^a^ | 3.48±0.02 ^c^ | 5.29±0.04 ^c^ | 1.89±0.06 ^b^ | 1.44±0.05 ^f^ | 0.19±0.07 ^cd^ |

**Table S2. The nutritional indices of SUPBs**

Note:Results are expressed as mean ± SD (n=3). Mean values with different lower-case letters in the same column showed significant differences at *p* < 0.0

**Table S3. Correlation analysis on 27 evaluation indicators**

|  | A_1_ | A_2_ | A_3_ | A_4_ | A_5_ | A_6_ | A_7_ | A_8_ | A_9_ | A_10_ | A_11_ | A_12_ | A_13_ | A_14_ | A_15_ | A_16_ | A_17_ | A_18_ | A_19_ | A_20_ | A_21_ | A_22_ | A_23_ | A_24_ | A_25_ | A_26_ | A_27_ |
| --- | --- | --- | --- | --- | --- | --- | --- | --- | --- | --- | --- | --- | --- | --- | --- | --- | --- | --- | --- | --- | --- | --- | --- | --- | --- | --- | --- |
| A_1_ | 1 |  |  |  |  |  |  |  |  |  |  |  |  |  |  |  |  |  |  |  |  |  |  |  |  |  |  |
| A_2_ | -.484** | 1 |  |  |  |  |  |  |  |  |  |  |  |  |  |  |  |  |  |  |  |  |  |  |  |  |  |
| A_3_ | -.979** | .593** | 1 |  |  |  |  |  |  |  |  |  |  |  |  |  |  |  |  |  |  |  |  |  |  |  |  |
| A_4_ | 0.32 | -.381* | -0.273 | 1 |  |  |  |  |  |  |  |  |  |  |  |  |  |  |  |  |  |  |  |  |  |  |  |
| A_5_ | -.858** | .718** | .885** | -.348* | 1 |  |  |  |  |  |  |  |  |  |  |  |  |  |  |  |  |  |  |  |  |  |  |
| A_6_ | 0.334 | -0.058 | -.373* | 0.139 | -.435* | 1 |  |  |  |  |  |  |  |  |  |  |  |  |  |  |  |  |  |  |  |  |  |
| A_7_ | -.499** | .440* | .502** | -0.122 | .481** | 0.147 | 1 |  |  |  |  |  |  |  |  |  |  |  |  |  |  |  |  |  |  |  |  |
| A_8_ | -0.263 | .555** | .392* | .395* | .402* | -0.045 | 0.023 | 1 |  |  |  |  |  |  |  |  |  |  |  |  |  |  |  |  |  |  |  |
| A_9_ | .543** | -.480** | -.485** | .914** | -.519** | 0.032 | -0.302 | 0.294 | 1 |  |  |  |  |  |  |  |  |  |  |  |  |  |  |  |  |  |  |
| A_10_ | -.893** | .726** | .899** | -.533** | .895** | -0.295 | .527** | 0.279 | -.723** | 1 |  |  |  |  |  |  |  |  |  |  |  |  |  |  |  |  |  |
| A_11_ | -.928** | .625** | .908** | -.556** | .905** | -.364* | .473** | 0.185 | -.738** | .978** | 1 |  |  |  |  |  |  |  |  |  |  |  |  |  |  |  |  |
| A_12_ | .629** | -.428* | -.574** | .655** | -.662** | 0.058 | -0.333 | 0.124 | .776** | -.637** | -.716** | 1 |  |  |  |  |  |  |  |  |  |  |  |  |  |  |  |
| A_13_ | -.814** | .480** | .864** | -0.061 | .669** | -.411* | .430* | .502** | -0.226 | .762** | .713** | -0.13 | 1 |  |  |  |  |  |  |  |  |  |  |  |  |  |  |
| A_14_ | 0.103 | -0.111 | -0.011 | .685** | -0.151 | -0.243 | -0.102 | .544** | .713** | -0.194 | -0.273 | .793** | .419* | 1 |  |  |  |  |  |  |  |  |  |  |  |  |  |
| A_15_ | -.894** | .450** | .884** | -.399* | .836** | -.426* | .378* | 0.096 | -.571** | .764** | .840** | -.750** | .583** | -0.313 | 1 |  |  |  |  |  |  |  |  |  |  |  |  |
| A_16_ | -.876** | .765** | .934** | -0.31 | .862** | -0.319 | .597** | .439* | -.505** | .920** | .878** | -.456** | .870** | 0.054 | .765** | 1 |  |  |  |  |  |  |  |  |  |  |  |
| A_17_ | -.841** | .540** | .876** | -0.087 | .913** | -.590** | .456** | .470** | -0.243 | .792** | .798** | -.361* | .821** | 0.214 | .751** | .842** | 1 |  |  |  |  |  |  |  |  |  |  |
| A_18_ | -0.27 | 0.17 | 0.215 | -.551** | 0.229 | -0.192 | 0.092 | -.396* | -.641** | .462** | .484** | -.350* | 0.089 | -.445** | 0.27 | 0.259 | 0.086 | 1 |  |  |  |  |  |  |  |  |  |
| A_19_ | -0.296 | .380* | 0.341 | 0.195 | .369* | 0.044 | .517** | .372* | 0.078 | 0.301 | 0.21 | 0.177 | .468** | .411* | 0.189 | .468** | .511** | -0.319 | 1 |  |  |  |  |  |  |  |  |
| A_20_ | -.915** | 0.321 | .906** | -0.16 | .816** | -.617** | .373* | 0.28 | -0.32 | .784** | .829** | -.399* | .858** | 0.157 | .806** | .799** | .928** | 0.19 | 0.333 | 1 |  |  |  |  |  |  |  |
| A_21_ | -.975** | .484** | .955** | -.392* | .896** | -.413* | .454** | 0.199 | -.582** | .874** | .925** | -.697** | .741** | -0.186 | .945** | .844** | .854** | 0.236 | 0.301 | .909** | 1 |  |  |  |  |  |  |
| A_22_ | -.735** | .735** | .825** | -0.098 | .691** | -0.235 | .500** | .649** | -0.269 | .793** | .706** | -0.187 | .905** | 0.325 | .513** | .915** | .740** | 0.149 | .428* | .683** | .638** | 1 |  |  |  |  |  |
| A_23_ | -.781** | .423* | .751** | -.624** | .662** | -0.211 | .348* | -0.142 | -.781** | .721** | .780** | -.806** | .433* | -.536** | .905** | .668** | .488** | .397* | 0.076 | .593** | .839** | .382* | 1 |  |  |  |  |
| A_24_ | -.902** | .513** | .891** | -.457** | .890** | -.453** | .384* | 0.126 | -.618** | .826** | .886** | -.730** | .621** | -0.281 | .969** | .798** | .810** | 0.264 | 0.297 | .832** | .968** | .536** | .890** | 1 |  |  |  |
| A_25_ | -.806** | .526** | .810** | -.406* | .692** | -0.139 | .448** | 0.079 | -.601** | .706** | .738** | -.660** | .536** | -0.316 | .898** | .761** | .580** | 0.225 | 0.329 | .615** | .839** | .524** | .929** | .886** | 1 |  |  |
| A_26_ | -.618** | 0.342 | .571** | -.770** | .536** | -0.17 | 0.244 | -0.325 | -.861** | .614** | .683** | -.857** | 0.217 | -.717** | .788** | .487** | 0.296 | .459** | -0.118 | .414* | .701** | 0.181 | .955** | .777** | .801** | 1 |  |
| A_27_ | -0.275 | 0.091 | 0.251 | 0.185 | .500** | -.357* | 0.071 | 0.232 | 0.131 | 0.176 | 0.246 | -0.127 | 0.119 | 0.118 | .345* | 0.179 | .577** | -0.226 | .499** | .427* | .376* | 0.007 | 0.076 | .435* | 0.198 | -0.034 | 1 |

Note: ** and * are significantly correlated at 0.01 and 0.05 levels (bilateral) respectively.

A1-A27: Water Content, Crude Fat Content, Crude Fiber Content, Crude Protein Content, Soluble Solid Content, P Content, Ca Content, Mg Content, K Content, Total Flavonoid Content, Pectin Content, Ascorbic Acid Content, Total Phenolic Content, Total Carotenoid Content, Total Sugar Content, Polysaccharide Content, Reducing Sugar Content, Soluble Sugar Content, Glucose Content, Fructose Content, Sucrose Content, Flesh “*L**” Value, Flesh “*a**” Value, Flesh “*b**” Value, Peel “*L**” Value, Peel “*a**” Value, Peel “*b*”*Value.

**Table S4. Rotation component matrix**

| Quality parameters | | Component | | | | |
| --- | --- | --- | --- | --- | --- | --- |
|  |  | 1 | 2 | 3 | 4 | 5 |
| A1 | Water Content | -0.90 | -0.32 | -0.14 | -0.06 | -0.14 |
| A2 | Crude Fat Content | 0.35 | 0.29 | 0.83 | -0.01 | 0.20 |
| A3 | Crude Protein Content | 0.91 | 0.24 | 0.25 | 0.05 | 0.15 |
| A4 | Crude Fibre Content | -0.13 | -0.83 | -0.08 | 0.30 | 0.16 |
| A5 | P Content | 0.75 | 0.35 | 0.44 | 0.31 | 0.01 |
| A6 | Ca Content | -0.60 | 0.07 | 0.10 | -0.14 | 0.67 |
| A7 | Mg Content | 0.42 | 0.15 | 0.12 | -0.08 | 0.70 |
| A8 | K Content | 0.27 | -0.46 | 0.75 | 0.20 | 0.04 |
| A9 | Flesh *L** Value | -0.30 | -0.87 | -0.16 | 0.24 | -0.05 |
| A10 | Flesh *a** Value | 0.77 | 0.43 | 0.42 | -0.10 | 0.08 |
| A11 | Flesh *b** Value | 0.79 | 0.50 | 0.30 | -0.02 | 0.00 |
| A12 | Peel *L** Value | -0.30 | -0.87 | -0.13 | -0.20 | -0.06 |
| A13 | Peel *a** Value | 0.91 | -0.18 | 0.25 | -0.18 | 0.12 |
| A14 | Peel *b** Value | 0.25 | -0.95 | 0.10 | -0.01 | -0.06 |
| A15 | Soluble Solid Content | 0.79 | 0.51 | 0.01 | 0.26 | 0.07 |
| A16 | Total Flavonoid Content | 0.84 | 0.20 | 0.43 | -0.08 | 0.23 |
| A17 | Pectin Content | 0.90 | -0.01 | 0.29 | 0.29 | -0.02 |
| A18 | Ascorbic Acid Content | 0.22 | 0.55 | -0.01 | -0.51 | -0.30 |
| A19 | Total Phenolic Content | 0.39 | -0.33 | 0.22 | 0.32 | 0.57 |
| A20 | Total Carotenoid Content | 0.97 | 0.06 | 0.03 | 0.13 | -0.09 |
| A21 | Total Sugar Content | 0.87 | 0.40 | 0.10 | 0.20 | 0.09 |
| A22 | Polysaccharide Content | 0.75 | -0.10 | 0.56 | -0.25 | 0.19 |
| A23 | Reducing Sugar Content | 0.60 | 0.73 | -0.07 | 0.05 | 0.18 |
| A24 | Soluble Sugar Content | 0.80 | 0.50 | 0.09 | 0.30 | 0.05 |
| A25 | Glucose Content | 0.66 | 0.52 | 0.04 | 0.15 | 0.36 |
| A26 | Fructose Content | 0.41 | 0.87 | -0.11 | 0.00 | 0.07 |
| A27 | Sucrose Content | 0.31 | -0.09 | 0.05 | 0.86 | -0.10 |
