# Supplementary material for: Quality assessment and variety classification of seed‐used pumpkin by‐products: Potential values to deep processing
Source: Food Sci Nutr. 2019 Nov 19;7(12):4095–104. doi: 10.1002/fsn3.1276 (PMC6924301; doi:10.1002/fsn3.1276)
Supplement: Supplementary file 3 [file FSN3-7-4095-s003.doc]

Fig S1. The pictures of one variety of pumpkin and ten varieties of seed-used pumpkins.
